# A Novel Framework for Arabic Dialect Chatbot Using Machine Learning

**DOI:** 10.1155/2022/1844051

**Published:** 2022-03-10

**Authors:** Nadrh Abdullah Alhassan, Abdulaziz Saad Albarrak, Surbhi Bhatia, Parul Agarwal

**Affiliations:** ^1^Department of Information Systems, College of Computer Science and Information Technology, King Faisal University, Alhasa, Saudi Arabia; ^2^Department of Computer Science and Engineering, Jamia Hamdard, New Delhi, India

## Abstract

With the advent of artificial intelligence and proliferation in the demand for an online dialogue system, the popularity of chatbots is growing on various industrial platforms. Their applications are getting widely noticed with intelligent tools as they are able to mimic human behavior in natural languages. Chatbots have been proven successful for many languages, such as English, Spanish, and French, over the years in varied fields like entertainment, medicine, education, and commerce. However, Arabic chatbots are challenging and are scarce, especially in the maintenance domain. Therefore, this research proposes a novel framework for an Arabic troubleshooting chatbot aiming at diagnosing and solving technical issues. The framework addresses the difficulty of using the Arabic language and the shortage of Arabic chatbot content. This research presents a realistic implementation of creating an Arabic corpus for the chatbot using the developed framework. The corpus is developed by extracting IT problems/solutions from multiple domains and reliable sources. The implementation is carried forward towards solving specific technical solutions from customer support websites taken from different well-known organizations such as Samsung, HP, and Microsoft. The claims are proved by evaluating and conducting experiments on the dataset by comparing with the previous researches done in this field using different metrics. Further, the validations are well presented by the proposed system that outperforms the previously developed different types of chatbots in terms of several parameters such as accuracy, response time, dataset data, and solutions given as per the user input.

## 1. Introduction

Every generation of the computer device is becoming more complex than the last one, and the troubleshooting task becomes more difficult for the end-user [1]. Computer problem detection is a complicated process that demands a high level of knowledge and skills. Troubleshooting is the process of locating the cause of a problem in a system and resolving it [2]. On average, computer repair technicians charge globally $60 per hour. Besides, the hourly rate can range from $40 to $90 per hour [3]. Some of these repairs can be addressed by providing assisting through a chatbot to the user. Furthermore, one of the remarkable artificial intelligence applications that have proven its efficiency recently is a chatbot. The chatbot is a simulating program that analyzes and processes human conversation, either written or spoken. It interacts with the end-users via digital devices as if users are communicating with a real person [4]. It provides 24-hour availability, instant answers, endless patience, personalization, and customized dialog to the end-user. Additionally, it extends to benefit various companies in this matter by saving the cost of hiring manpower, achieving customer satisfaction, and reaching new customers [5]. Chatbot relies heavily on Natural Language Processing (NLP), commonly used to make human spoken language understandable for computer machines [6]. The goal of NLP is to take the unstructured input and produce a structured representation of the text that contains understandable language for textual chatbot conversation [7]. Also, Natural Language Processing is known as a model trained which belongs to an Arabic chatbot's textual input and output [8]. The essential part of building chatbots is the conversational interfaces. Since the chatbot responds in terms of text to the user query, NLP yields an adequate textual conversation for the chatbot [9]. In this paper, we rely on NLP to process Arabic language by using the presented framework. The research has identified and minimized the gaps existing in the field of dialects in Arabic language and the advancements in the chatbots by comparing the performance of the proposed system with the previously developed researches.

The rest of this paper is organized as follows: next, we present motivation and contributions and a tiny backdrop. We discuss the challenges for developing Arabic chatbots. We then present comparison with existing systems and describe our framework. After that, we illustrate chatbot mechanism used in this paper including experimental result and a preliminary user evaluation.

### 1.1. Motivation and Contributions

The motivation behind this work has portrayed the fact that Arabic chatbot does not reach the required limit in terms of response speed, dataset type, and how the chatbot handles user input. The outcome of this paper will lie in the implementation based on industrial applicability in building an Arabic Chabot that is capable of assisting end-users to solve troubleshooting problems in Arabic. This framework incurs repair costs by an expert troubleshooting system. It will aid to diagnose IT troubleshooting and solve technical issues within a few seconds. The contributions of this research work are given as follows:

- To study the background on previously developed chatbots and the challenges of using Arabic chatbot in multiple domains;
- to propose the novel IT troubleshooting chatbot framework for Arabic language;
- to create the dataset from various sources including multiple companies such as Microsoft, HP, Samsung, Huawei, and different websites that address common IT problems/solutions and apply preprocessing tasks for further uses;
- to compare the proposed chatbot with previous researches on varied dialogues, writing the parameters;
- to evaluate the proposed chatbot by comparing it with other chatbots on different functional evaluation metrics;
- to develop a service-based chatbot to provide idiomatic solutions for common IT problems that can be considered for practical industrial applications.

## 2. Background

The revolution of information technology during our era predetermines that all governmental and private sectors need rapid progress of digital transformation and mounting development. Our lives are not devoid of using technologies, especially those that involve artificial intelligence techniques. Technology has gathered the world societies in a common cyber environment, where every technology that emerges in any place spreads to all applicable societies.

Artificial intelligence relies on data as input; then it is processed via specific approach to generate the demand output like-human thinking. Even though, there is a concern whether AI application will rescind the need of human thinking gradually. In fact, AI works as extension of human brain to solve complex problems and process huge amount of data at the same time. One of AI techniques used with different language by machine learning is the acknowledgment of city names. The promising study application areas in the realm of postal automation are the recognition of handwritten city names. For recognition, use a nonsegmentation method (Holistic approach). The role of the convolutional neural network (CNN), which is one of the deep learning techniques, is deconstructed in this paper [8], detecting hand signs, by anticipating the next word or recommending the most relevant word, and then generating the word that deaf persons communicate with people using sign language.

### 2.1. Arabic Chatbot

Before time, chatbot was described as a text-interaction between human and machine learning by simulating an online conversation (chat) with a user in natural language. Chatbot was first introduced in 1996. Precisely, ELIZA was the first chatbot created in MIT, which arguably came close to imitating a human reply. ELIZA was given an input sentence and it identifies keywords and patterns to match those keywords against a set of preprogrammed rules to generate appropriate responses [10]. Late in 1971, Kenneth Colby at Stanford created PARRY, a preprogrammed chatbot act-like schizophrenia diagnosed patient and was able to express fears, anxiety, and beliefs. By the time, chatbot's discoveries increased in 1995 by Richard Wallace who created A.L.I.C.E chatbot, using English conversation patterns in AIML files such as (<category>…<pattern></pattern>….</category>). AIML is a subordinate of web extensible mark-up language XML. After that, during the millennium, many forms of chatbot were invented. To get a sense, all millennium chatbot inventions refer now to modern chatbots in the AI industry.

The Arabic language is considered as one of the Afroasiatic languages. Due to built-in alphabetical structure, it is a notoriously difficult language in categories of programming text processing, because of reading and writing direction, from right to left, which is the opposite direction of English language. To further complicate things, the form of Arabic letter is totally different than English language and vowels are omitted from written Arabic. Globally, there are 422 million Arabic speakers around the world [11]. According to a British council report, Arabic language took the second place for each native speaker who lived in northern Africa such as Algeria and Morocco and western Asia such as Georgia and Azerbaijan and took the first place at Arabian Peninsula such as Saudi Arabia and United Arab Emirates. Moreover, it took the 4th place as a useful language in the trade market in the UK and Arabic countries as well [12].

In this respect, significant work has been done on chatbots. However, the Arabic language is rarely used in chatbot especially in the IT sector. Additionally, few numbers of Arabic chatbots applications/websites are available for the end-user. For instance, in educational domains, Nabiha, one of Arabic chatbots, is concerned about helping college students using informal Arabic via automatic conversation with student inquiries [13]. On the other hand, BOTTA is a fun Arabic conversational chatbot aiming at using an Egyptian accent to have fun chat with users [14]. Also, Quran chatbot is an Arabic one answering users' inquiries religiously, by generating extracted replies from the Holy Quran [15]. Also, Tafsir Al-Ahlam is an Arabic robot specialist interpretation of dreams using a smart search engine to generate output from a local database of a wide range of interpretations taken from the books attributed to Ibn Sirin and Nabulsi son of Shaheen and other accredited authors in the Islamic heritage books, following the origins and rules of interpretation of dreams from the Quran and the Hadith [16]. In medical domain, NALA is an application that provides medical consulting under the supervision of the Ministry of Health [17].

Customers may use service-oriented chatbot to find information on big, complex domains that are difficult to navigate. This is recognized as “service-based chatbot;” the main reasonability for this chatbot is to provide a service, contrary to other chatbots acting as only an entertainer. Many users find it difficult to find the details that they need from website search engine results due to the site's abundance of data. During a conversation with the user, the service-oriented chatbot serves as an automated customer service agent, providing natural language responses and more targeted information. This virtual agent is also programmed to assist with general IT-related questions [18].

### 2.2. Chatbot Framework

A framework is a term commonly used in IT software development which is referred to as a structure consisting of many phases that work together as a foundation for software developers to build programs for a specific platform [19]. As it follows by bot framework, a set of processes and requirements guide the developer while he/she is programming the bot as well as solving any potential problems met by the developer at the same phase. Bot framework provides tools, services, and skills needed which facilitates the developer's job [20].

Most frameworks share certain fundamentals phases, but they differ according to the purpose of creating the framework itself, based on the most proposed framework that is currently going on in the field of chatbot development. A five functional phases are listed as follows. Automatic speech recognition (ASR) is a technology that provides a voice identifier with a computer interface through the human voice that allows a human being to interact with a computer in such a way that it seems very close to the real human. Natural language understanding (NLU) is highly connected to machine reading comprehension. The process of selecting parts of sentences and analyzing the meaning is tedious because the machine needs to determine the correct syntactic structure and semantics of the language used. Dialogue management is mainly responsible for coordinating the various components of a chatbot. Natural language generation is mainly relating to the process of retrieving and producing the answer. Lastly, there is text to speech. The answer is ready to be retrieved for the end-user [21]. The following is a brief description of some frameworks used for chatbots in Arabic.

### 2.3. Comparison with Existing Systems

The first framework is the framework of an intelligent Arabic chatbot for teaching Islamic history. The main goal of this framework is to help and guide designers for the efficient use of chatbots and simulations for teaching Islamic history. It consists of three phases explained as follows.

#### 2.3.1. Interaction

This phase includes the user input such as query, and it is stored immediately in the Heroku cloud server. This framework adopts a cloud computing server to avoid standard dedicated server's problems and costs.

#### 2.3.2. The Processing Phase

This phase includes the use and implementation of many programming languages for handling, storing, processing, searching, and retrieving the data. After that, determining the size of storage might be needed since the content of Islam history is too large to be handled on smartphone's capacity. So, the framework presents the MongoDB technique to be used in this phase.

#### 2.3.3. Learning Phases

This phase enables the bot to be recordable and learnable, by sorting user input/query at bot memory. The program manager will update the bot's memory by matching words or queries with the appropriate answer. Finally, the last process is using some advanced programs such as neurolinguistic programming (NLU), and it can be implemented manually by human review or automatically by NLU. The current NLU program cannot support Arabic language but it can be replaced with any suitable text processing techniques [22].

The second framework is Facebook Arabic chatbot based on deep learning using RASA framework aimed at answering student inquiries at the University of Islam in Indonesia via Facebook message. College students often need immediate information like asking for something to help desk, especially during this COVID-19 pandemic, due to Facebook popularity in Indonesia. There were 166.500.000 Facebook users in Indonesia in August 2020, which accounted for 60.8% of its entire population. Thus, RASA framework has been developed to match with Arabic content [23].

To construct an Arabic chatbot, most of the existing research relies on third-party framework platforms likewise PANDORABOT platform. As a result, some difficulties in dealing with Arabic letters have arisen as well as HTML tags, database scope, response, and input processing. The following framework will overcome these gaps by using programming Python groundwork. In terms of Arabic content, the framework uses AI to improve NLP and machine learning techniques; see Table 1.

As shown in Table 1, most of the Arabic chatbot applications used premade platform like Pandorabots API. This kind of platform has subscribed package followed by yearly/monthly fees. The developer is in charge of renewing the subscription fee. Eventually, the developer gets exhausted with unnecessary additional costs and limited features, gaining complexity at run time. This conceded on the top list of solutions provided by the proposed framework. Also, code-based application frameworks are rarely supported with Arabic content. This research focuses on solving the problems of complexity and user-friendly programming support with effective applications.

## 3. The Proposed Methodology

**Figure 1:**
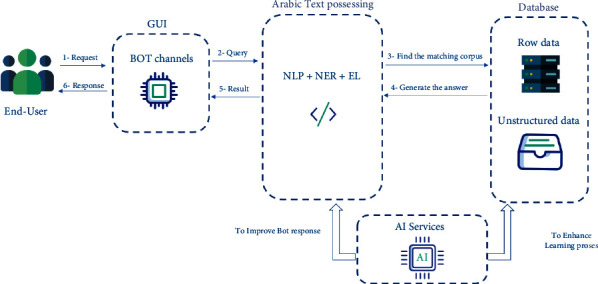
System proposal framework of devolving an Arabic troubleshooting chatbot to diagnose and solve technical issues.

This research introduces a novel IT chatbot framework for troubleshooting supporting Arabic language. The framework comprises four phases: GUI, Arabic text processing, AI services, and database.  Figure 1 demonstrates the components and the processes involved in the framework design.

The chatbot starts by receiving the input as “text format” from the end-user. The bot channels transfer the question/inquiry regarding any potential IT troubleshooting as a query to the Arabic text processing phase. At this stage, the framework examines the match corpus from the existing knowledge in the database phase and extracts the answer as result to the end-user. Along with that, get the benefit from AI services to enhance the learning process in the database phase for nonexisting knowledge and improve generally text process in Arabic text processing phase. Text processing phase not only provides NLP, but also provides named entity recognition (NER) by labelling named “real-world” objects, like persons, companies, or locations. Entity linking (EL) removes the uncertainty of meaning from an ambiguous sentence, phrase, or other linguistic units. Text processing is useful for more than just decoding and analyzing text back to its origins. Rather, it goes above and above to generate data that aids in the usage of chatbot measures. As it is a real number, it is very easy to do statistical and arithmetical calculations towards NLP, making it suitable for use in statistical ML models and finally using bot channels to retrieve the solution in GUI format through a friendly interface. Moreover, one of the goals intends to contribute to the NLP uses of the Arabic language. The proposed framework endeavor deeply presents how to process Arabic text in the IT domain.

Moreover, every chatbot has its dataset or in other terms corpus. The Quran chatbot is one of the Arabic chatbots that adapted the Java program to produce an Arabic AIML dialogue corpus [15]. BOTTA is another Arabic application using AIML files through the Pandora's platform to generate a dialogue corpus [14]. However, there is no Arabic chatbot that has been adopted in Python programming language and generates the corpus via Python environment. So, we built a service-based chatbot to provide idiomatic solutions for common IT problems using python. Besides, build a knowledge base of IT troubleshooting, which can be reused by other researchers.

### 3.1. Chatbot Builder

The work has been implemented on a personal laptop with specific features. To deal with the capabilities of the chatbot system, CPU, which is a Dual-Core Intel Core i7 with 8 GB RAM, is used. The processing speed of the CPU is essential in the programming and data mining phases. The mean programming language used is Python and any related library/package is used to run the chatbot mechanism.

### 3.2. Dataset

The incredible amount of data on the Internet is a rich resource for any chatbot application. Web scraping is the process used in this project to transform unstructured data into a specific classification, thus to extract data using web scraping with Python including nltk library to reprocessing Arabic content. And use an open-source project corpus for the Saudi dialect called MADRA PROJECT by data analysts from Jordan. A corpus is a selection of different input statements and responses for the bot to practice with. This project allows Arabic developers to use more than 26 Arabic dialects [24]. But, here in this project, we build our corpus called chattest.text, with the benefit of merging MADRA's corpus and our corpus.

### 3.3. Arabic Text Handling

By using online 50 contents as database source, whether structured as csv or unstructured as text/yml, some problems occur. Thus, we apply text preprocessing with nltk: tokenization, by splitting the Arabic text into smaller pieces or “tokens.” **Normalization** aims to put all Arabic content in one level. Noise removal cleans up the text from extra white spaces.

Tables 2 and 3 list the details on the dataset and websites used for extracting the data. In total, 50 texts have been retrieved with nested content, with difficulty in forming data according to the classification prepared previously in the research and some python's library/package, not supporting Arabic language. But by using core web sm for the latest version and scan of the HTML tag of required content, use the HTML attribute to specify the content.

**Figure 2:**
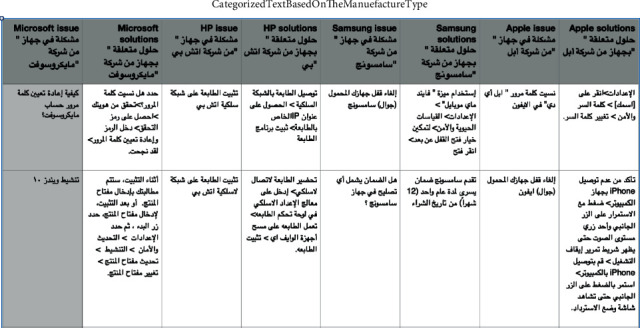
Final form of data scraped after running the code categorized.

**Figure 3:**
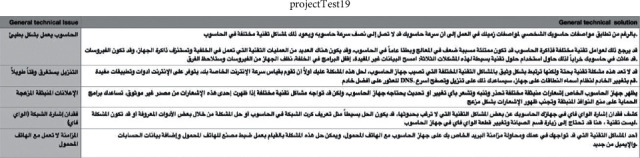
Final form of data scraped after running the code categorized.

The result of data after applying the classification process is shown in Figures 2 and 3.

### 3.4. Chatbot Mechanism

After generating a dataset that is suitable for the IT problem/solution domain, the implementations were done using Python programming language. Python is more suited to make changes to an existing legacy system and offers different methods/libraries such as NumPy, pandas, PyBrain, and SciPy that help expedite AI development. For example, you can leverage proven libraries like scikit-learn for ML and use regularly updated libraries like Apache MXNet, PyTorch, and TensorFlow for DL and bots' projects that support text possessing in Arabic field.

Thus, we create a greeting message for the chatbot and then create (AI keyword matching) for the chatbot's corpus. We use (AI) intelligent search through the corpus using spacy and Python environment. Keyword matching works on using a keyword that appears in the query. And it identically matches any word in a chatbot corpus. In a basic retrieval system, keyword matching cannot be functional without having the full sentence or query to retrieve the relevant answer. But, by using AI keyword matching, the chatbot does not analyze the whole user input but focuses on searching words on phrases defined in the user says [25]. The best way to explain the behaviour of AI keywords is to use a realistic example as shown in Table 4. In case the chatbot cannot find the entry that matches a keyword, it will return: “I'm sorry! I do not understand you.”

## 4. Experimental Results

The proposed framework is evaluated in terms of effectiveness. The comparison of the chatbot developed is compared with other chatbot's platforms using different parameters including time of response, type of dataset, and dealing with user input. The two versions of the chatbot are developed, one by using the proposed framework and the other by using an external framework/platform which is Pandorabot [26]. Both chatbots were fed by the same datasets. To measure the variation between the two bots, different experiments have been performed.  Table 5 shows the type of dataset used, time of response, and how to process the inputs and then illustrate the question chosen to test both chatbots. After that, there are the result and findings.

## 5. Evaluation

Goal-oriented chatbots are a form of currently popular chatbot whose main function is to assist users with a specific set of tasks, such as “how can I unlock my Samsung (mobile) device?” Some publications with goal-oriented chatbots conducted evaluations that were related to the chatbot's objectives. The metrics include the number of successful conversations ended by the system, percentage of the problem solved, average dialogue length, and average of user utterance length [27].

The participants comprised 6 participants from the different educated background like engineering, linguistic, and finance background. Two were females, 4 were males, 3 were aged between 25 and 34, and 2 were aged between 35 and 44. All participants who participated were not having any IT background.

### 5.1. Goal-Oriented Chatbots Evaluation for “المساعد الذكي” Chatbot

We have presented the key findings of designing an Arabic chatbot unescorted by any third-party platforms, based on our proposed framework. The components of our framework are crafted thoughtfully; each phase can add/upgrade your bot mechanism into AI chatbot using the Arabic language.

**Figure 4:**
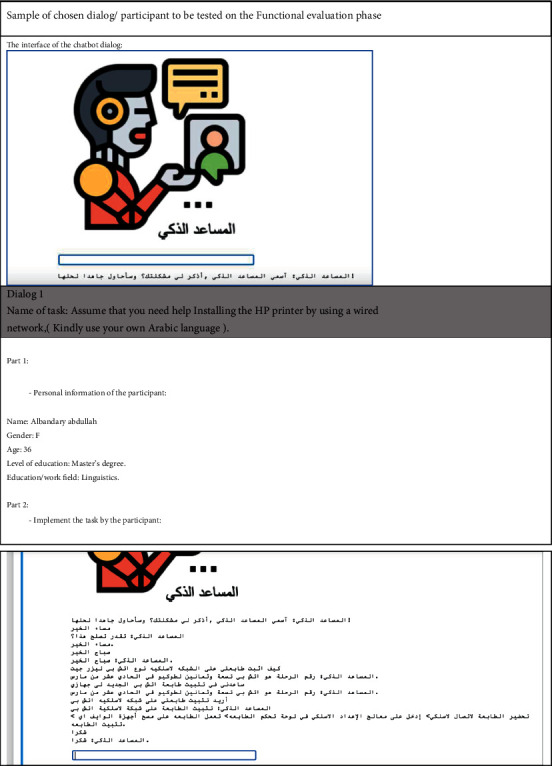
Goal-oriented chatbots evaluation for “المساعد الذكي” chatbot.

Most of the current Arabic chatbots use the basic dialog (Q/A), third-party platforms, and complex AIML files [28]. However, we found that our framework directs the developer to get leverage of AI open-source package. In this project, we adopt a Python library to preprocess over 50 Arabic common IT problems/solutions and trained the bot to diagnose the problem (input), then generating the solution (output) as shown in Figure 4.

### 5.2. Performance Measures

The performance of our chatbot has been tested through experiments performed between chatbots, developed from zero starting point, and the other has been tested by using an external platform which is Pandorabot, Arabic troubleshooting chatbot [29, 30]. Both were suited to have any type of Arabic data (formal/informal) without having strict inputs to a specific form. The analysis is shown in Table 6.

**Figure 5:**
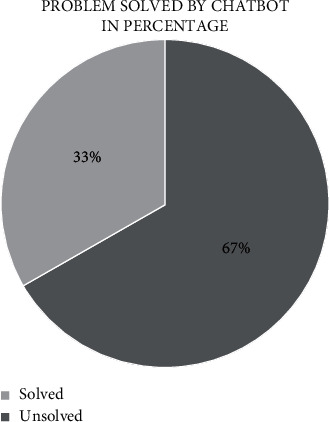
Solved/unsolved cases presented in pie chart.

**Figure 6:**
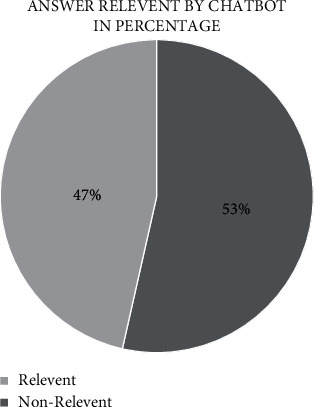
Relevant/nonrelevant chatbot's answer presented in pie chart.

The bot uses AI keyword matching to analyze user input and match it with the most relevant problem/solution. AI keyword matching is a Python package deal [31, 32] with Arabic letters intelligently, needlessly to afford subscription fees to third-party platforms for creating an Arabic chatbot. Figures 5 and 6 show the solved cases in terms of evaluating the performance of the proposed chatbot.

Another testing method of “المساعد الذكي” chatbot is using goal-oriented chatbots evaluation. The result shows that 67% of the total problems are solved successfully and the average dialogue length is around 13 sentences per/conversation. Moreover, the average user utterance length is 5 sentences per/conversation. People usually speak over 8–10 sentences in one minute. Thus, chatbot's conversation does not exceed one minute from the end-user to receive useful information as shown in Table 7. The performance has been evaluated using metrics [33].

## 6. Conclusions

In this paper, an introductive framework for devolving Arabic troubleshooting chatbot to diagnose and solve technical issues is developed. A framework that supports Arabic text for better machine's understanding is implemented. The framework takes advantage of AI to enhance NLP and machine learning techniques in terms of Arabic content. This chatbot is a service-based chatbot. Thus, 10 websites were scanned to gain 50 problems and their reliable solutions. Chatbot evaluation shows that after comparing two previous built Arabic chatbots on the same dataset the proposed troubleshooting chatbot is capable of diagnosing and answering users' input more effectively. It is more convenient for the users to use their own accent (informal Arabic language) and receive the required answer.

For the evaluation techniques, this research classified them into two main categories, stated as comparison of the chatbot produced by following the proposed framework and others on chatbots platform. The evolution of the chatbot is done by the real users using functional evaluation matrices. Despite the findings of the research, there are some limitations because the majority of Arabic chatbot application has limited type of question answering (QA) corpus via NLP. To make interaction with the Arabic language easier, libraries for programming languages must be developed.

This knowledge is an important basis for future Arabic chatbot development. The framework is developed using the latest released package of Python and Arabic language, by leveraging a variety of AI including keyword matching techniques. However, the Arabic language branched out into special grammar, character, and forms that may be difficult for the Python language to identify in this research. The findings can be used as a guide for potential chatbot developers to choose appropriate evaluation methods for their chatbots based on their design, domain, aim, and evaluation intent. Also, it is possible to add voice bots by using voice-to-text recognition after incorporating the required technology. Furthermore, the outcomes of chatbot evaluations could be compared through various chatbots that were evaluated independently.

## Figures and Tables

**Table 1:** Comparison of a few of the relevant works pertaining to the Arabic chatbots.

Ref no.	Motivation	Datasets used	Obtained accuracy	Limitations

[13]	This article introduces Nabiha, a new Arabic dialect chatbot dedicated to assisting college students	The datasets collected are 248 inputs/outputs from the KSU IT students' accounts in Askme.com. The methods used in this work are premade platform which is called PANDORABOT platform	The result of this paper shows that 15.38% of the total answer is not accurate, 53.85% is somewhat accurate, and 30.77% is very precise	The dataset should be expanded; we need to address the issue of HTML tags as well as Twitter's text area constraints
[14]	BOTTA's goal is to create a conversational environment and connect with as many Arab users as possible. She's the first chatbot to speak in an Arabic dialect, which helps her achieve her goal of amusing people who are used to conversing in the language	BOTTA is using AIML and launched it on the PANDORABOT platform	BOTTA's pattern matching will be able to correct 85.1 percent of the spelling errors observed in spontaneous Arabic typing	Using corpus-based machine learning approaches, BOTTA's pattern matching has to be improved. Further development will involve morphological analysis of the input and experiments with lemma-based pattern matching using existing tools for Egyptian Arabic processing

**Table 2:** Some of the open-source websites with the common companies for IT troubleshooting.

Name of company	Name of the website	Does it provide Arabic content?

Microsoft	https://support.microsoft.com/ar-sa/windows/windows-update-%D8%A7%D9%84%D8%A3%D8%B3%D8%A6%D9%84%D8%A9-%D8%A7%D9%84%D9%85%D8%AA%D8%AF%D8%A7%D9%88%D9%84%D8%A9-8a903416-6f45-0718-f5c7-375e92dddeb2	Yes
HP	https://support.hp.com/emea_middle_east-ar	Yes
Samsung	https://www.samsung.com/sa/support/mobile-devices/how-do-i-solve-the-problem/	Yes
Apple	https://support.apple.com/ar-sa	Yes
Huawei	https://consumer.huawei.com/sa/support/	Yes

**Table 3:** Some open-source websites used to gather data for IT troubleshooting.

Name of the website	Does it provide Arabic content?	Does it need translation?

https://trendsarab.com/post/4385/%D9%85%D8%B4%D8%A7%D9%83%D9%84-%D8%AA%D9%82%D9%86%D9%8A%D8%A9-%D8%B4%D8%A7%D8%A6%D8%B9%D8%A9	Yes	No
https://www.arageek.com/tech/how-to-deal-with-windows-problems	Yes	No
https://www.youm7.com/story/2019/6/26/8-%D9	Yes	No

**Table 4:** Presentation of an example of an Arabic dialogue implemented by the chatbot.

Dialogue in Arabic
“المساعد الذكي: آسمي المساعد الذكي,أذكر لي مشكلتك؟ وسأحاول جاهدا لحلها!”
“المستخدم: أريد إزالة تحديث في جهازي”
Here, the user uses only 2 words that match with chatbot corpus: “إزاله” and “تحديث” and still the chatbot diagnoses the problem by saying “إزالة تحديث مثبت في ويندز”
“المساعد الذكي: إزالة تحديث مثبت في ويندز:
And retrieve the most relevant answer:
“لا نوصي بإزالة أي من التحديثات التي تم تثبيتها ومع ذلك، إذا كان من الضروريّ إزالة أحد التحديثات، فيمكنك القيام بذلك في محفوظات التحديثات”
Translation of the dialogue
Chatbot: hi, my name is “the smart assistant” tell me your technical problem? And I will try to help you
User: I want to remove an update on my device
Chatbot: we do not recommend removing any installed updates. However, if it is necessary to remove an update, you can do it in the update history.”

**Table 5:** Comparison of the chatbots (our chatbot and Pandorabot).

Comparison criteria	Chatbot produces by using our proposal framework	Chatbot produces by using Pandorabot platform

Type of dataset	- Use unstructured Arabic data (text/yaml). And there is no need to define each question/answer because our chatbot can search through all corpus within seconds to retrieve the data.	- Use AMIL file required to define each question as <pattern></pattern> and the answer as <template></template> for each dialog. - The question must be in capital letter (English case). - The data must not have any kind of punctuations because the platform will ignore it immediately and will not deploy it.
Time of response	The answer was retrieved immediately.	The answer took around 30 seconds to retrieve, and it could be “apologize message.”
Dealing with user input	- The user can insert any Arabic input by using the formal and informal Arabic language.	- The user can insert only the exact question already fixed in the AIML file. Except that, the chatbot will ignore the question and retrieve “apologize message.”
The experimentation	We insert the same dataset for both bots. And we use the same question format. The question used is “نسيت كلمة مرور جوالي الايفون” and the answer should be “نسيت كلمة مرور” ابل أي دي “في الايفون:الإعدادات>انقر على [اسمك] > كلمة السر والأمن > تغيير كلمة السر” which contained diagnosis of the problem followed by the solution.	
The result	- If the question is in informal Arabic language, the bot retrieves the correct answer. Input: “نسيت كلمة مرور جوالي الايفون” Output: the bot retrieves the correct answer.	- If the question is in informal Arabic language, the bot retrieves “apologize message.” Input: “نسيت كلمة مرور جوالي الايفون” Output: the bot retrieves error “apologize message.” - If the question is in an exact AIML file premade, the bot retrieves the correct answer. Input: نسيت كلمة مرور “ابل أي دي” في الايفون Output: the bot retrieves the correct answer.
Finding	- Our chatbot was capable of having any type of Arabic data without having strict inputs to a specific form. The bot uses AI keyword matching to analyze user input and match it with the most relevant problem/solution.	- Pandorasbot working as simple chatbot was capable of matching a text string and offering an answer only when the exact sentence match is found.

**Table 6:** General analytics of goal-oriented evaluation part 1.

Dialogs	Problem solved (yes = 1, no = 0)	Problem solved (first reply = 1/second reply = 0,1/more than 2 replies = 0)	Answer relevant (yes = 1, no = 0)

D1	1	0	1
D2	1	0.1	1
D3	0	0	0
D4	1	1	1
D5	1	1	1
D6	0	0	0

**Table 7:** General analytics of goal-oriented evaluation part 2.

Dialogs	Dialogue length (in sentence/line)	User utterance length (in sentence/line)	Number of times in which the participant paraphrases the question

D1	16 sentences	5 sentences	3 times
D2	13 sentences	4 sentences	1 time
D3	17 sentences	8 sentences	4 times
D4	11 sentences	4 sentences	0 times
D5	7 sentences	3 sentences	0 times
D6	14 sentences	7 sentences	3 times
The average	13.17	5.17	1.8

## Data Availability

This paper is the result of an ongoing project; the dataset is developed and cannot be released at this stage.
